# A possible superconductor-like state at elevated temperatures near metal electrodes in an LaAlO_3_/SrTiO_3_ interface

**DOI:** 10.1038/s41598-018-29945-y

**Published:** 2018-08-01

**Authors:** Taeyueb Kim, Shin-Ik Kim, Sungjung Joo, Sangsu Kim, Jeehoon Jeon, Jinki Hong, Yong-Joo Doh, Seung-Hyub Baek, Hyun Cheol Koo

**Affiliations:** 1 Center for Electromagnetic Metrology, Korea Research Institute of Standards and Science, Daejeon, 34113 Korea; 2 Center for Electronic Materials, Korea Institute of Science and Technology, Seoul, 02792 Korea; 3 Department of Display and Semiconductor Physics, Korea University, Sejong, 30019 Korea; 4 Center of Spintronics, Korea Institute of Science and Technology, Seoul, 02792 Korea; 5 Division of Nano & Information Technology, KIST School, Korea University of Science and Technology, Seoul, 02792 Korea; 6 Department of Physics and Photon Science, Gwangju Institute of Science and Technology (GIST), Gwangju, 61005 Korea; 7 KU-KIST Graduate School of Converging Science and Technology, Korea University, Seoul, 02841 Korea

## Abstract

We experimentally investigated the transport properties near metal electrodes installed on a conducting channel in a LaAlO_3_/SrTiO_3_ interface. The local region around the Ti and Al electrodes has a higher electrical conductance than that of other regions, where the upper limits of the temperature and magnetic field can be well defined. Beyond these limits, the conductance abruptly decreases, as in the case of a superconductor. The samples with the Ti- or Al-electrode have an upper-limit temperature of approximately 4 K, which is 10 times higher than the conventional superconducting critical temperature of LaAlO_3_/SrTiO_3_ interfaces and delta-doped SrTiO_3_. This phenomenon is explained by the mechanism of electron transfer between the metal electrodes and electronic *d*-orbitals in the LaAlO_3_/SrTiO_3_ interface. The transferred electrons trigger a phase transition to a superconductor-like state. Our results contribute to the deep understanding of the superconductivity in the LaAlO_3_/SrTiO_3_ interface and will be helpful for the development of high-temperature interface superconductors.

## Introduction

The interface formed between a LaAlO_3_ (LAO) film and TiO_2_-terminated SrTiO_3_ (STO) has a wide range of electronic phases, including superconductivity^1^, ferromagnetism^2^, and spin-orbit interactions^3–5^. These exotic phenomena open new opportunities to study fundamental issues in condensed matter physics, and the LAO/STO interface may be useful for future applications, such as field effect transistors^6–9^. Although LAO and STO have an insulating nature, the electrically conducting channel formed between them provides high-mobility carriers and even shows a superconducting behavior that is tunable by adjusting the gate voltage^10^. This phase is classified as having *interface superconductivity*^11^ and shows spatial inhomogeneity on the micrometric scale^12–14^ and coexistence with the ferromagnetic phase^12,13^. Despite many efforts to determine the origin of this interface superconductivity over the course of the past decade, these observations are still the subject of debate.

The atomic *d*-orbitals of titanium (Ti) ions in a LAO/STO interface play a crucial role in electronic transport^15^. Their energies are split into different energy levels due to a crystal field^15–17^. The lower level orbitals are normally occupied by an intrinsic electronic transfer process, the so-called ‘polar catastrophe mechanism’^18–20^, and by electrons that come from oxygen vacancies near the interface^21–23^. Several approaches to establish an extrinsic electronic transfer process have been tested to control the population of these *d*-orbitals. Application of a gate voltage in a field effect transistor geometry induces an additional high-conducting carrier that can come from the occupation of the high energy *d*-orbitals^4,24^. Insertion of external dopants into the interface changes the electric population of these orbitals. A variety of studies have been performed on doping: superconductivity is weakened by Cr doping^25^, electronic transport is not significantly affected by Tm and Lu doping^26,27^, electron density and mobility are quenched by Mn doping^28^, and the magnetic order is not affected by Ru doping^29^. However, it is difficult to find evidence for the enhancement of carrier mobility due to doping in these reports. Recently, a more effective method has been suggested to control the population of these *d*-orbitals: adjusting the charge transfer between the conducting channel and the metal overlayer deposited on the LAO surface^30,31^. In particular, Ti and Al were tested as a metal overlayer and an increase in carrier density was experimentally observed, implying a tunable occupation of the *d*-orbitals^32,33^. Even for an insulating LAO/STO interface, an Al overlayer enables the formation of a conducting channel by this charge transfer process^34^.

In this study, we also attempted to perform electron transfer from Ti (or Al) to an LAO/STO interface. Instead of a metal overlayer on a LAO surface as used in previous studies, we deposited Ti (or Al) electrodes after removing (or breaking) the LAO layer, which allowed for direct access of the electrons in these metals to the LAO/STO interface. Although the method of *d*-orbital occupation is similar to that of previous studies, the results are quite different; we observed a high conducting state in this local region, and its conductance varied dramatically with the temperature and magnetic field, indicating a type of phase transition. This new phase has a well-defined threshold temperature and magnetic field reminiscent of a superconductor. Furthermore, this threshold temperature is approximately 4 K, which is 10 times higher than the conventional critical temperature of superconductors in the conducting channels of LAO/STO and delta-doped STO. Thus, the observed states are clearly distinguishable from the superconducting phases that have been reported thus far. We believe that these results are due to the Lifshitz transition^35–37^: i.e., the electrodes give electrons to Ti^+3^ ions in the LAO/STO interface, and these excess electrons occupy the high energy *d*-orbitals and trigger a phase transition to a superconductor-like state.

## Results

**Figure 1:**
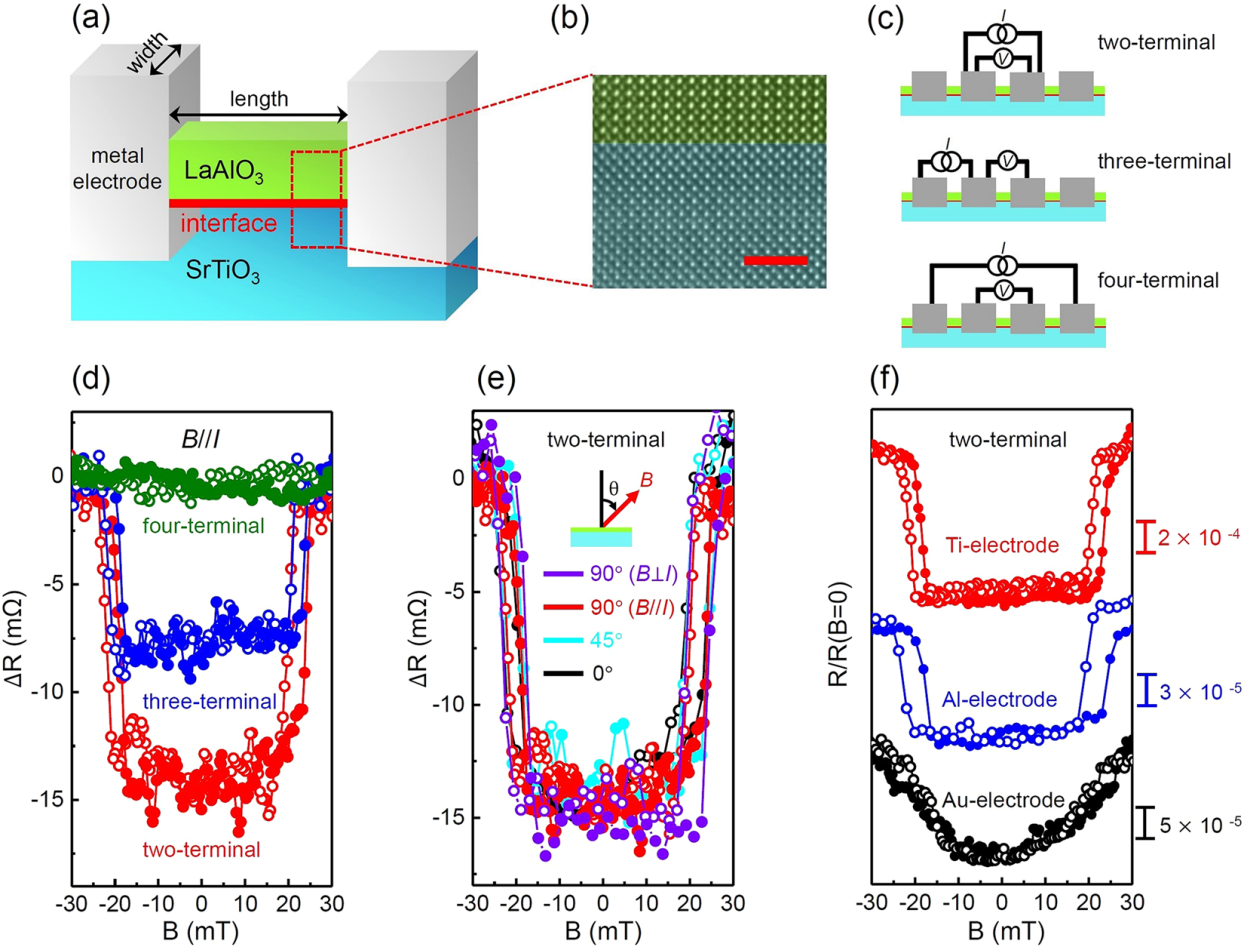
Device structure and magnetoresistance. (**a**) Schematics of a conducting channel at the LAO/STO interface. (**b**) A high-resolution transmission electron microscopy image taken near the channel. The scale bar is 2 nm. (**c**) Illustrations of three measurement methods: two-, three-, and four- terminal configurations. Magnetoresistance of a Ti - electrode sample (sample 1) at 1.8 K (**d**) for the three configurations and (**e**) for various magnetic field directions. (**f)** Normalized magnetoresistance at 1.8 K for three types of metal electrodes. For the two- and three- terminal configuration, the Ti- and Al- electrode samples show an abrupt change in resistance near the field of 20 mT regardless of the field direction. However, there is no abrupt change in the magnetoresistance curve for the Au-electrode sample. The data in (**e**,**f**) were obtained using the two-terminal configuration, and θ designates the direction of magnetic field, *B*, such that θ = 0° indicates the direction perpendicular to the interface. The data in (**f**) are shifted for clarity. *B* // *I* and *B* ⊥ *I* represent a field direction of θ = 90°, which is parallel or perpendicular to the current direction, respectively. ${\rm{\Delta }}R\equiv R(B)-R(B=30\,mT)$. In (**d**) *R*(*B* = 30 *mt*) = 19.145 Ω, 2.915 Ω and 13.321 Ω for two-, three- and four-terminal configurations, respectively. In (f) *R*(*B* = 0) = 19.13 Ω, 177.80 Ω and 12.06 kΩ for the Ti-, Al- and Au- electrode samples, respectively.

Four samples were prepared in this work: two Ti-electrode samples, called sample 1 and sample 2, and two individual samples with Al and Au electrodes. Sample 1 and the Al and Au electrode samples were fabricated using the same LAO/STO wafer. After depositing metal electrodes near the LAO/STO interface and allowing electron transfer in the metal into the conducting channel, the electrodes were also used as electric terminals for current flow and voltage measurements. In this study, channel resistance near an electrode is distinguished from a resistance which was not affected by the electrode. ‘*Internal resistance*’ is the term used in this paper to describe resistance free from the influence of the electrodes, and it represents certain intrinsic properties of the conducting channel in a LaAlO_3_/SrTiO_3_ interface. Accordingly, the ‘*internal region*’ refers to the part of the channel that is far enough from the electrodes to not be affected by them. The resistance was measured using a DC current source and a voltmeter. To perform measurements near the electrodes, we collected signals using various measurement configurations: two-, three-, and four-terminal configurations, as depicted in Fig. 1(c). For the two-terminal configuration, the measured resistance included the local resistance near the two electrodes as well as the internal resistance. For the three-terminal configuration, the local resistance near a single electrode was measured, excluding the internal resistance, while only the internal resistance was measured for the four-terminal configuration. In the three-terminal configuration, the middle electrode, as shown in the second panel in Fig. 1(c), acted as both the current and voltage terminals and the right voltage probe has non-zero distance from the middle electrode. This finite distance allowed the signal from this configuration to contain a voltage drop on the local LAO/STO channel near the middle electrode. Naturally, the resistance obtained from this three-terminal configuration involved the contact resistance that came from the junction of two different materials, i.e., LAO/STO and the metallic electrode. Additionally, this three-terminal signal contained the channel resistance of the LAO/STO interface near the electrode. In particular, the Ti and Au electrodes were designed to be wide and close to each other; the length and width of these electrodes shown in Fig. 1(a) are 5 *μ*m and 400 *μ*m, respectively. This design makes the local resistance around the electrodes account for a great part of the measured signal. Measurements were performed in a temperature-controllable system. In order to search for some enhanced superconductor-like properties at elevated temperatures, we focused on a temperature range that was higher than the critical superconducting temperature of the internal region.

Figure 1(d) shows the magnetoresistance of the Ti electrode sample for the three measurement configurations at 1.8 K with a magnetic field *B* parallel to the electric current. The resistance was constant for the four-terminal configuration, which implied that the internal region was not affected by the magnetic field. For the other two cases, the resistance increased abruptly when the field magnitude exceeded 20 mT. The resistance change was approximately 7.5 mΩ for the three-terminal configuration, and this value doubled for the two-terminal configuration. The number of electrodes related to local effect in the two- and three-terminal configuration were two and one, respectively. Thus, these data indicate that the magnetic field only affects the local region near the electrodes (not the internal region) and that this phenomenon occurs equally for each electrode.

This resistance change was independent of the direction of the magnetic field. We varied the field direction with respect to the interface and current direction. In Fig. 1(e), all of the measured curves from different field directions were merged into a single curve. Thus, the conducting state of the system was only influenced by the field magnitude, regardless of its direction. It was unusual that the magnetoresistance was constant with respect to the magnetic field direction because the electronic orbitals around the LAO/STO interface had strong directional properties because they were sandwiched between two different insulators, LAO and STO. Thus, they had no degrees of freedom to move perpendicular to the interface. If the magnetoresistance is governed by the Lorentz force, it should be dependent on the field direction, which is inconsistent with the observed curves in Fig. 1e. Additionally, the resistance in our device increased at the critical magnetic field, but in typical ferromagnetic materials, the magnetoresistance decreases with increasing field strength when the magnetic field is applied perpendicular to the bias current. Thus, anisotropic magnetoresistance, which is related to ferromagnetism, can be ruled out in our results. The anomalous Hall effect also depends on the magnetization direction and is not applicable to our system. Ruling out the Lorentz force and magnetism, a suitable candidate for the causative mechanism is superconductivity. In a superconductor, the magnetic field exceeding a critical value breaks the superconductivity, which makes the resistance abruptly increase. This critical field is usually independent of the field direction, as in the present cases^38^. In this connection, it is speculated that our samples have a high conducting local phase near the electrode and that this phase has a definite upper limit of the magnetic field, as does the superconductivity.

One feature of magnetoresistance is its high dependence on the metallic element of the electrode. As shown in Fig. 1(f), the samples fabricated with Ti or Al electrodes had a superconductor-like magnetoresistance. However, the Au electrode sample did not show an abrupt jump in resistance; the resistance versus field curve was quadratic, displaying a typical ordinary magnetoresistance caused by the Lorentz force^39^. These different behaviors for various metallic electrode were related to the work function of the metal, which governs the occupation of electronic orbitals in the LAO/STO interface. We discuss this issue later. The difference in signal strength between the Ti and Al electrode sample was attributed to the different fabrication processes of these two metals on the LAO/STO interface (see the Methods section for details). The relatively high disorder around the electrode was accompanied by the fabrication of Al, which resulted in a small signal in the Al electrode sample in comparison with the Ti electrode sample.

**Figure 2:**
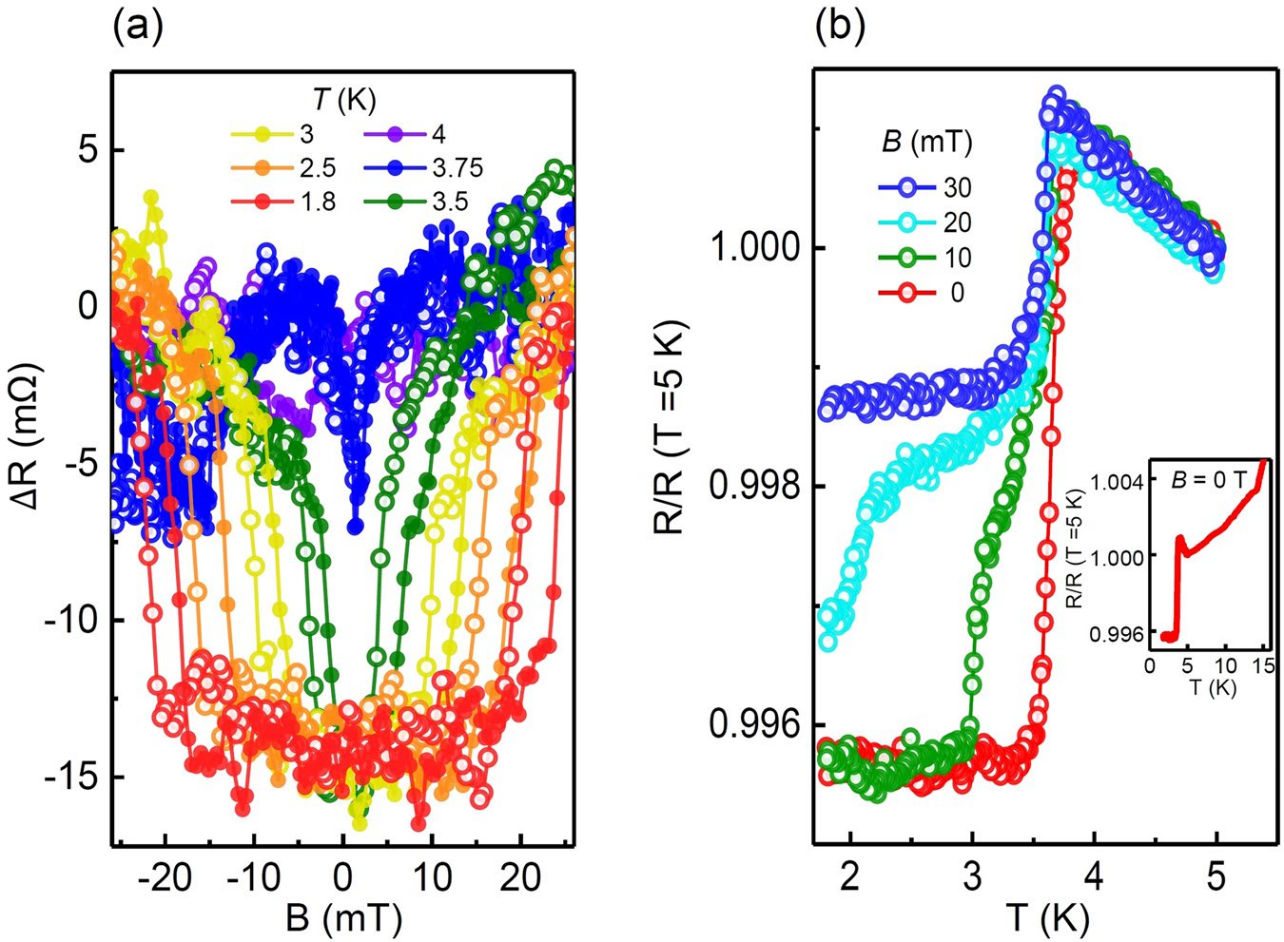
Temperature dependence of transport properties. (**a**) Magnetoresistance curves for various temperatures in a Ti-electrode sample. (**b**) Resistance as a function of temperature under several magnetic fields. The inset in (**b**) shows data for an extended temperature range. Both (**a**,**b**) were measured for sample 1 using the three-terminal configuration with *B* // current. A threshold magnetic field can be defined where the magnetoresistance curve changes abruptly, and this value decreases with increasing temperature. Similarly, a threshold temperature can be defined where the resistance shows an abrupt jump, and this value is 3.75 K in the absence of magnetic field, as shown in (**b**). *R* (*T* = 5 K) in (**b**) is 2.92 Ω.

The apparent jump in resistance at a specific magnetic field for the Ti and Al electrode samples allows for a clear definition of a threshold magnetic field, indicating a boundary between the low and high conducting states. The high conducting state below the threshold magnetic field shows a dramatic variation with temperature *T*. As shown in Fig. 2(a), the high-conducting region in the magnetic field becomes narrower with an increase in temperature. A threshold magnetic field of 22 mT at 1.8 K decreases to 5 mT at 3.5 K, and finally, the high-conducting state disappears at approximately 4 K. Thus, the system has a threshold temperature above which the high-conducting state does not exist regardless of the magnetic field strength. The threshold temperature is clearer in Fig. 2(b), where the resistance as a function of temperature is shown. In the absence of a magnetic field, the resistance jumped at 3.7 K, indicating a threshold temperature, and this threshold temperature decreased with an increasing magnetic field.

The well-known superconductivity in the conducting channel of the LAO/STO interface arises in the internal region (far from the electrodes) and is distinguishable from the underlying high-conducting state near the electrodes in this work. The data shown in Fig. 2(b) bear a similarity to those of internal superconductivity^40^ except for the resistance offset. A significant difference between them is the characteristic temperature. The critical temperature of the internal superconductivity has been reported to be several hundred millikelvins, while the threshold temperature of the present sample reached 3.7 K. The inset in Fig. 2(b) shows the temperature dependence of the resistance above the threshold temperature, which has the well-known characteristics of the LAO/STO interface: a metallic behavior above 10 K and Kondo effect^41^ from 3.75 K to 10 K. The Kondo effect, a clear upturn of the resistance with decreasing temperature, is caused by the interplay between the carrier localization and mobile charge. Brinkman *et al*.^2^. observed this effect in LAO films grown under high oxygen pressure, and Han *et al*.^42^ analyzed this effect in connection with localized Ti^3+^ ions and oxygen pressure. The Kondo temperature of our sample was 10.43 K, which can be described well by a model proposed in ref.^43^. Above the threshold temperature, our sample showed the temperature dependence of a conventional non-superconducting channel in the LAO/STO interface. Below the threshold temperature, however, the internal region was still in a non-superconducting state, while the region near the electrode was in a superconductor-like state. Therefore, compared to a conventional superconductor, the measured resistance in Fig. 2(b) does not decrease to zero.

**Figure 3:**
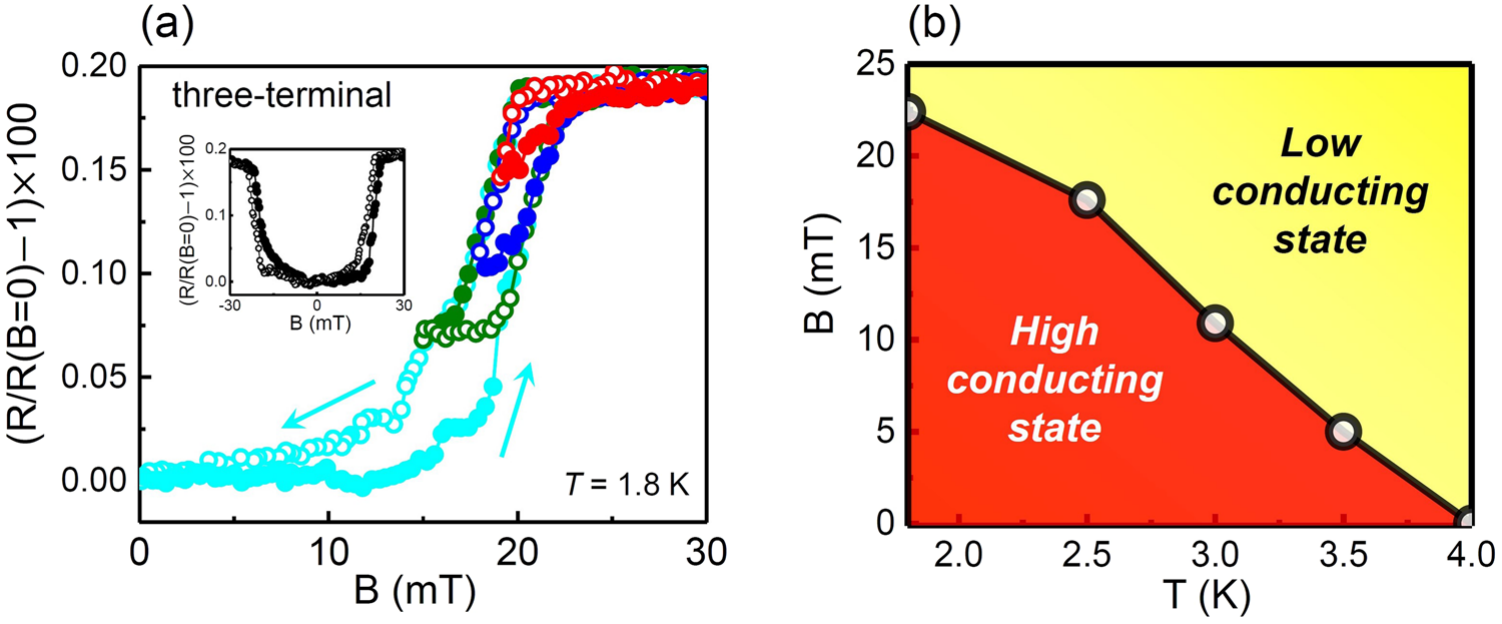
Hysteresis and phase diagram. (**a**) Various hysteresis loops in magnetoresistance curves along with the transition between the high/low conducting states for sample 2. The solid circles are the data collected as the magnetic field was increased, and the open circles are the data collected as the magnetic field was decreased. The inset shows the case of a full field sweep between −30 mT and +30 mT. (**b**) Phase diagram of the two conducting states in the temperature and magnetic field plane for sample 1. The boundary between them is the threshold temperature or threshold magnetic field.

Hysteresis of physical quantities is normally accompanied by a phase transition and is one of the most interesting phenomena observed in the LAO/STO system. Many researchers have insisted that hysteresis in magnetoresistance is related to the ferromagnetic phase^2,24,40^. All of the magnetoresistance curves of the Ti and Al electrode samples showed hysteretic behaviors, a shift of the measured resistance along the sweep direction of the magnetic field. The observed hysteresis was reproducible and independent of the field-sweep rate in the range below 0.1 *mT*/s. Thus, the present hysteresis does not originate from a time-dependent transient nature^36^. Figure 3(a) shows the systematic behavior of the hysteretic magnetoresistance in our sample. Despite different historical paths produced by the sweeping control of magnetic field, all of the curves are merged into one of two curves: one is the response to an increasing field and the other to a decreasing field, which are typical characteristics of a rate-independent hysteresis associated with irreversible thermodynamic changes, such as phase transitions. Generally, when an external parameter such as a magnetic field causes a phase transition of the system, the natural inertia inherent in the system restrains the system from triggering the phase transition, which results in a hysteretic behavior. Therefore, the hysteresis shown in Fig. 3(a) confirms that our system undergoes a phase transition between two different states. Although the curves illustrate a minor loop and memory effect, further study is necessary to clarify whether this hysteresis is related to ferromagnetism.

A phase diagram differentiating the high- and low-conducting states near the electrodes is depicted in Fig. 3(b). The overall shape was similar to the phase diagram of the internal region^44^. However, there was a 10-fold difference in the physical quantities between them. The threshold temperature was 10 times greater than the critical temperature of the internal region, and the threshold magnetic field was 10 times less than the critical field of the internal region. This scaling tendency suggests that the observed phase transition in this work may be an extension of the internal one to the limit of a high-temperature and low-magnetic-field regime.

**Figure 4:**
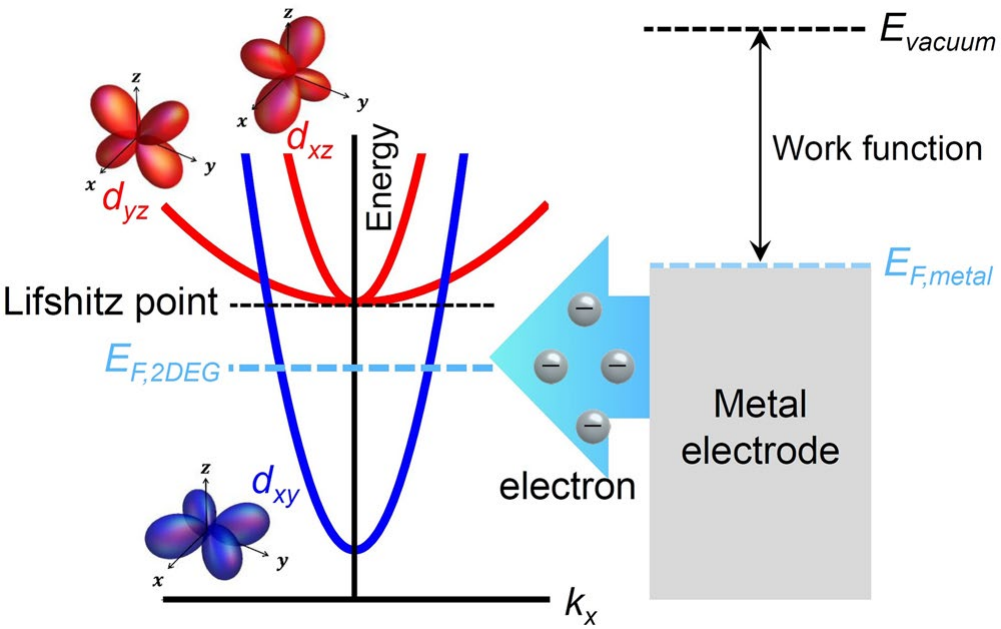
An illustration of electron transfer between the LAO/STO interface and a metal electrode. Schematic energy band of the *d*-orbitals in Ti ions near the conducting channel in an LAO/STO interface (left panel)^35,37^ and the work function of a metal electrode (right panel). The *dyz* and *dxz* orbitals have higher energy than the Fermi level of the channel, *E*_*F,2DEG*_. For an electrode with small work function such as Ti and Al, the Fermi level difference causes electrons to be transferred to the channel, and the electrons occupy the *dyz* and *dxz* orbitals. A Lifshitz transition occurs when *E*_*F,2DEG*_ passes through the Lifshitz point and the *dyz* and *dxz* orbitals begin to be occupied. For a simple view, the energy band and the Lifshitz point are depicted assuming the absence of spin-orbit coupling. With spin-orbit coupling, the *dyz* and *dxz* orbitals are strongly coupled and form a hybrid orbital, *dyz/dxz*^35^. Two Fermi levels, *E*_*F,2DEG*_ and *E*_*F,metal*_ in this diagram, show the state before electron transfer occurs between the channel and the metal electrode.

It has been widely accepted that superconductivity in a LAO/STO interface is related to the emergence of high-mobility carriers^45,46^. We believe that the observed high-conducting phase can be attributed to the electric population of the high-mobility states near the Ti (or Al) electrodes, which are rare in the internal region. Electronic transport properties come from the *d-*orbital electrons in Ti^+3^ ions at the TiO_2_-terminated interface. Only a small fraction of those electrons contribute to electrical conductance, and most of them reside in localized states fixed to Ti ions because of strong Hubbard and nearest-neighbor Coulomb interactions^47^. Among the *d-*orbitals, *d*_*xy*_, *d*_*xz*_, and *d*_*yz*_ are responsible for the electric current, and their schematic energy diagrams are shown in Fig. 4. The energy of *d*_*xy*_ is lower than the energy of *d*_*xz*_ and *d*_*yz*_ because of the asymmetric confinement potential at the interface^15–17^. Thus, carriers in a LAO/STO interface can be classified into two types: high-density electrons residing in the *d*_*xy*_ state and low-density high-mobility electrons occupying a hybrid state, *d*_*xz*_/*d*_*yx*_, combined with *d*_*xz*_ and *d*_*yz*_^45,48–50^. Carries in the *d*_*xz*_/*d*_*yx*_ states are known to be engaged in superconductivity^4,51–53^. Under ordinary conditions, the *d*_*xz*_/*d*_*yz*_ orbitals are rarely occupied; however, the system is ready to move to high-mobility states when electrons are supplied to occupy these states.

A number of studies have investigated ways to populate *d-*orbitals. It has been theoretically and experimentally confirmed that metals with low-work functions deposited atop an LAO/STO structure can supply carrier electrons to the LAO/STO interface across the LAO layer^30,31^. The low-work function relative to that of STO causes a Fermi level difference, and electrons are transferred from the metal toward the STO to align the Fermi level on both sides. The work functions of Ti and Al are 4.3 and 4.2 eV, respectively, and are especially effective for this carrier transfer process^33,34^: they increase the carrier density of the interface channel by more than 6 × 10^13^ *cm*^−2^ ^32^. In this study, the Ti and Al electrodes supply electrons to the interface channel, as in the previous studies mentioned above (refer to the schematic in Fig. 4). For more effective electron transfer, however, we removed the barrier, i.e., the LAO layer, and made the electrodes directly contact the interface channel. Our results are quite different from those of previous studies: there is clear evidence of the high-mobility electronic state arising from the charge transfer and a dramatic phase change of this state similar to a superconducting transition. This effective doping process provides excess electrons to the *d*_*xz*_/*d*_*yz*_ orbitals and triggers a superconductor-like transition. The abrupt conductance variation observed in this study can be attributed to the Lifshitz transition^35,37^, which occurs when the carrier density exceeds a critical value and new orbitals are populated. In the region far from the Ti deposition layer, the system lies below a critical carrier density and most electrons reside in the *d*_*xy*_ states. Near the Ti or Al electrodes, however, the supply of excess electrons makes the Fermi energy enter the *d*_*xz*_/*d*_*yz*_ orbitals. This transition is also very sensitive to temperature and magnetic fields, providing a clear definition of the threshold temperature and magnetic field. According to the above scenario, a metal electrode with a high work function cannot induce such a transition. The work function of Au is approximately 5.3 eV, which is high enough relative to the STO’s electron affinity, 4.1 eV. Therefore, electron transfer from the Au electrode to STO does not take place and the *d*_*xz*_/*d*_*yz*_ orbitals in the STO cannot be populated. We used a gold electrode, and as expected, the results shown in Fig. 1(f) do not indicate any sign of a transition to a high conducting state.

## Discussion

Since the concept of interface superconductivity^11^ was introduced 50 years ago^54^, ground-breaking works on insulator interfaces have been conducted, leading to the discovery of a superconducting phase at LAO/STO interface in 2007^1^. Our results suggest the reasonable possibility of dramatically enhancing this superconductivity in terms of the critical temperature and magnetic field. The phase diagram obtained in this work covers the temperature and magnetic field regime, which has not been reported in previous research^44^. In addition to the superconducting phase, coexistence with the ferromagnetic phase in LAO/STO interfaces is an emerging issue^12,13^. The systematic hysteresis behavior of magnetoresistance in this study suggests the coexistence of these two phases because hysteresis of magnetoresistance in LAO/STO interfaces is considered to be a sign of a ferromagnetic phase^2,24,40^. Furthermore, inhomogeneity of conductance is another important issue in LAO/STO interfaces^12–14^, the origin of which is still an open question. We also observed a strong local nature exhibiting high conductance near metal electrodes and elucidate the origin of this inhomogeneity by the effective doping process induced by electron transfer from low-work-function electrodes. For engineering purposes, this effective doping method can be used to pattern a conducting channel in the LAO/STO interfaces^34^, which may lead to a noble nanoscale fabrication technology. Thus, our results may be major step toward a fundamental understanding and future device applications of interface superconductivity in an LAO/STO structure.

## Methods

We grew a LAO layer on a TiO_2_-terminated STO (001) single crystal substrate using pulsed laser deposition. The substrates were connected to a resistive heater and positioned 5 cm from the target. A KrF excimer laser beam (wavelength of 248 nm) was focused on a LAO single-crystal target with an energy density of 1.5 J/cm^2^ at 2 Hz. The LAO thin films were grown at a substrate temperature of 700 °C with 1 mTorr of oxygen pressure. The thickness of the deposited LAO film was five-unit cells. The carrier density and mobility of the LAO/STO interface were, respectively, 2 × 10^13^ *cm*^−2^ and 900 *cm*^2^/*V·s* at 1.8 K in the as-grown condition. The epitaxial growth of a LAO layer on a STO substrate was confirmed via a cross-sectional high-resolution transmission electron microscopy image (see Fig. 1(b)). The metal electrodes were fabricated using two methods: Ti or Au electrodes were made by deposition using a DC sputter and Al electrodes were made by wire wedge bonding. Ti or Au was deposited on the LAO/STO interface after the LAO layer was removed by Argon ion-beam etching. This etching was performed in a RF plasma system operated at low pressure (2×10^−3^ Torr) with a power of 20 W and a discharge voltage less than 100 V. During this process, etching and deposition were performed without vacuum breaking. We took resistance measurements for the exposed regions of the Argon ion-beam and confirmed that those regions were not electrically conductive. The junction size of each Ti (or Au) electrode was 400 × 400 *μm*^2^, and the length between individual electrodes was approximately 5 *μm* (refer Fig. 1(a)). The junction size of the Al electrode was approximately 50 × 100 *μm*^2^, and the length between them was 500 *μm*. The resistance was measured using the standard DC technique: a DC current was applied by a Keithley 6221 current source, and the voltage drop was measured by a Keithley 2182 A nano-voltmeter. These instruments had a high enough output and input impedances, 10^14^ Ω and 10^10^ Ω, respectively, to ensure a stable pair of source and meter. The voltmeter had a resolution of 1 nV with a digit of 6.5, which provided enough precision to discern resistance changes in our samples. To reduce possible noise, we repeated measurements more than 50 times for each data point and performed a numerical average of them.
